# Ethnic Disparities in Glioblastoma Markers: Impact of Chromosome 7 Gain and 10 Loss Alterations on Clinical Survival Outcomes

**DOI:** 10.32604/or.2026.077076

**Published:** 2026-05-21

**Authors:** Fang-Ying Chiu, Yun Yen

**Affiliations:** 1 Department of Medical Imaging and Radiological Sciences, College of Medicine, Tzu Chi University, Hualien, Taiwan; 2 Center for Cancer Translational Research, Tzu Chi University, Hualien, Taiwan; 3 Center for Brain and Neurobiology Research, Tzu Chi University, Hualien, Taiwan; 4 Graduate Institute of Oncology, College of Medicine, National Taiwan University, Taipei, Taiwan; 5 TMU Research Center for Cell Therapy and Regeneration Medicine, Taipei Medical University, Taipei, Taiwan; 6 Ph.D. Program for Cancer Molecular Biology and Drug Discovery, College of Medical Science and Technology, Taipei Medical University, New Taipei, Taiwan

**Keywords:** Biomarker, chromosome 7 gains and chromosome 10 losses (chromosome +7/−10), epidemiology, ethnicity, glioblastoma, mortality, precision medicine

## Abstract

**Objective:** Glioblastoma (GBM) is the most common primary malignant brain tumor and is characterized by significant intratumoral heterogeneity. This study aimed to investigate the clinical and genomic landscapes of GBM across diverse ethnic populations to identify potential prognostic markers. **Methods:** Leveraging The Cancer Imaging Archive (TCIA) and bioinformatics modeling, White, African, and Asian American cohorts were analyzed. Patients were stratified according to the 2021 WHO classification of central nervous system (CNS) tumors. Population-specific genomic drivers and phenotypic markers were evaluated for their impact on outcomes. Survival rates across age, sex, and ethnicity were estimated using the Kaplan-Meier method and Cox proportional hazards models. **Results:** Incidence was highest among White American males aged 50–60 (*n* = 105, 34%) compared with females in the same age group (*n* = 48, 24%). Asian Americans exhibited a lower incidence rate (0.46-fold) compared to White individuals. In the adjusted model, Asian American ethnicity showed a trend toward a different survival outcome (HR 0.464; 95% CI 0.206–1.047; *p* = 0.064). The co-occurrence of chromosome 7 gains and chromosome 10 losses (+7/−10) was associated with significantly lower survival rates compared to those without combined alterations (HR 0.798; 95% CI 0.647–0.984; *p* = 0.035). **Conclusions:** Epidemiological evidence-based medicine shows that the chromosome +7/−10 genotype predicts poor survival, particularly in high-risk White (Caucasian) males. Its lower prevalence in African and Asian American cohorts suggests the presence of distinct ancestry-specific oncogenic drivers. Integrating ethnic stratification into clinical frameworks is essential for improving biomarker-informed diagnostics and ensuring equity in personalized GBM treatment strategies based on ancestry-informative genetic markers.

## 1 Introduction

Glioblastoma (GBM) is the most common primary brain cancer in adults, with an incidence of 3.23 cases per 100,000 people, accounting for approximately 14.3% of all brain tumors and 49.1% of all malignant central nervous system (CNS) tumors according to the Central Brain Tumor Registry of the United States (CBTRUS) [1,2,3,4,5]. In epidemiology, GBM is the most common malignant brain tumor accounting for 14.3% of CNS tumors [6]. The 2021 World Health Organization (WHO) classification of CNS tumors includes updates reflecting substantial progress in the classification and treatment of gliomas. It introduced molecular diagnostic criteria for isocitrate dehydrogenase (IDH)-wildtype tumors and included specific molecular markers for the diagnosis of GBM, such as chromosome 7 gain and chromosome 10 deletion (+7/−10), transcriptase (TERT) promoter mutations, and epidermal growth factor receptor (EGFR) amplification [7].

Neuro-oncology treatment has made progress with advances in immunotherapy and precision medicine. However, ethnicity and genomic alterations in epidemiological disparities in GBM mortality remain a persistent challenge [8,9]. Notably, divergence in clinical outcomes and mortality from this aggressive tumor has been attributed to socioeconomic and genetic risk factors, including limited access to predictive, prognostic screening, advanced stage of brain tumors at diagnosis, and a higher prevalence in male patients [10,11,12,13]. Exceptionally, assigning molecular heterogeneity in the microenvironment of gliomas and classifying them into distinct clinical groups based on codeletion of the short arm of chromosome 1 (1p) and the long arm of chromosome 19 (19q) (1p/19q), IDH mutations, and telomerase reverse *TERT* promoter mutations, which are characterized by different mechanisms of pathogenesis. Advances in molecular genetics have led to more accurate diagnosis and prognosis of CNS tumors. Not only can it serve as a predictive or prognostic marker to support precision medicine, but it can also identify different patient subpopulations most likely to benefit from a predetermined treatment, serve as a potential biomarker, or even suggest a surrogate endpoint. More pertinently, despite efforts to identify influencing factors, a series of studies investigating the use of non-invasive imaging biomarkers and the provenance of The Cancer Imaging Archive (TCIA) such as qualitative vs. quantitative image analysis, Translational Radiomics Imaging Biomarkers (TRIB^®^) via data-driven extraction to expand and understand horizons in different diseases [14,15,16]. Furthermore, predicting the prognosis of treatment response and survival outcomes that underlie decisive ethnic differences in the setting of genomic alterations remains limited.

GBM has a poor survival rate due to recurrence and resistance to existing treatment modalities which highlights the importance of interactions between glioma cancer cells and cells of the tumor microenvironment (TME). Cancer stem cells and immune cells play key roles in the TME of glioma, including perivascular, hypoxic, and invasive niches, and their roles are evolving as our understanding of the cellular players involved deepens. Understanding targeted immunotherapy and elucidating the diverse interactions between tumor cells are crucial for advancing novel precision strategies such as neoantigen vaccines, ultimately leading to improved patient outcomes. Current classifications lack routine clinical use, and their generalizability to non-European GBM populations remains unverified. It remains underexplored whether the findings from these studies translate to GBM patients of other ancestries.

This bioinformatics study aims to characterize molecular signatures and genomic alterations associated with ancestral disparities in the incidence and survival of IDH-wildtype GBM. By identifying these signatures across diverse populations, this work establishes a framework for diagnostic and prognostic biomarkers to facilitate clinical translation.

## 2 Materials and Methods

### 2.1 Study Design, Cohort Selection and Genetic Signature

**Figure 1:**
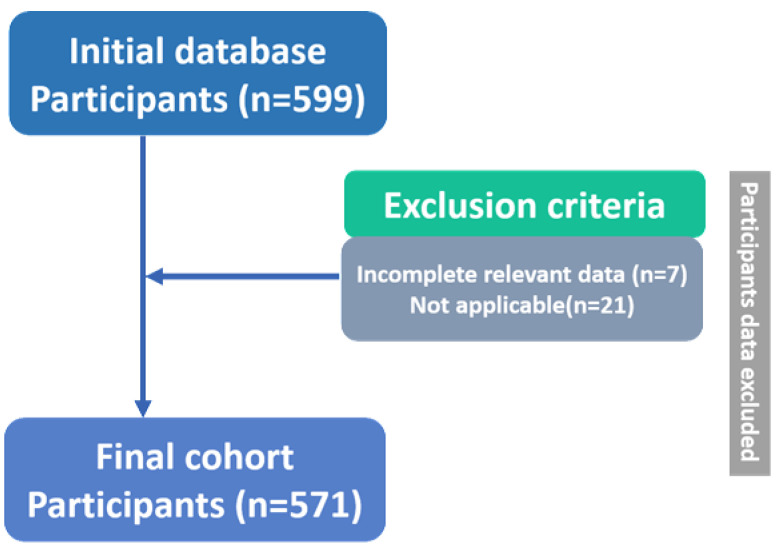
Flowchart of patient enrollment and selection. Initial population (*n* = 599), exclusion criteria (*n* = 28), final population (*n* = 571) for the primary analysis.

This retrospective cohort profile used GBM patient data from the TCIA publicly available dataset retrieved in 2021 from public repository (http://doi.org/10.7937/K9/TCIA.2016.RNYFUYE9). This dataset has previously been utilized to investigate the molecular characterization of GBM [14,15] as well as to explore phenotype features from the cancer genome atlas research network [17,18,19,20]. The population of interest included identification of ethnicity (i.e., White, African, and Asian Americans) with a first-time pathologic diagnosis of GBM (*n* = 599). Exclusion criteria included patients with incomplete relevant data (*n* = 7) or those with variables categorized as not applicable (*n* = 21) in Fig. 1. The analytic cohort enrolled criteria on ethnicity, sex, age, genomic alteration-related, i.e., both gain of entire chromosome 7 and loss of the entire chromosome 10 (+7/−10) information and overall survival (OS) was calculated as the time in months from the date of initial histological diagnosis to the date of death or last follow-up (Table 1). The final de-identified population consisted of 571 patients. This study was approved by the Institutional Review Board of Hualien Tzu Chi Hospital (No. IRB110-007-B) in accordance with the tenets of the Declaration of Helsinki. The IRB waived the requirement for informed consent due to the study’s retrospective design and the use of de-identified data.

**Table 1:** Demographic and clinical characteristics of GBM cohort stratified by ethnicity^a^.

Attribute/Characteristic	White American	African American	Asian American	*p*-Value^c^
**Sex**				0.504
Male^b^	309 (61.0)	31 (60.8)	10 (76.9)	
Female^b^	198 (39.0)	20 (39.2)	3 (23.1)	
**Sociodemographics**				0.199
Age at diagnosis, Median (IQR)	59 (48.0–67.0)	53 (43.5–67.0)	52 (38.5–65)	
**Age group at diagnosis**				0.095
<50^b^	118 (23.3)	20 (39.2)	6 (46.1)	
50–60^b^	153 (30.2)	13 (25.5)	3 (23.1)	
61–70^b^	135 (26.6)	8 (15.7)	3 (23.1)	
71–80^b^	79 (15.6)	10 (19.6)	1 (7.7)	
81–90^b^	22 (4.3)	0 (0)	0 (0)	
**Chromosome 7 gain/chromosome 10 loss**				<0.001**
Chromosome +7/−10^b^	323 (64%)	30 (59%)	4 (31%)	
No combined CNAs^b^	164 (32%)	14 (27%)	9 (69%)	
Unknown^b^	20 (4%)	7 (14%)	0 (0%)	
**Karnofsky performance status**				0.39
<70^b^	87 (17.2)	11 (20.8)	3 (23.1)	
≥70^b^	288 (56.8)	21 (40.0)	9 (69.2)	
Unknown^b^	132 (26.0)	19 (39.2)	1 (7.7)	

Note: Bold denotes key variables identified for the sensitivity analysis, including sex, sociodemographics, age group at diagnosis, chromosome 7 gain/chromosome 10 loss, Karnofsky performance status. ^a^The cohort reflects random data for each participant. ^b^Values are presented as (*n*%), unless otherwise specified. ^c^*p* < 0.001 (**) is considered as statistically significant. CNAs: copy number alterations; IQR: interquartile range.

### 2.2 Ethnicity Ascertainment

Clinical and demographic data for the TCGA-GBM cohort were retrieved from The Cancer Genome Atlas Glioblastoma Multiforme data collection (https://www.cancerimagingarchive.net/collection/tcga-gbm/) accessed in 2021. Following standardized TCGA protocols, race and ethnicity were determined via patient self-report during clinical enrollment at the contributing Tissue Source Sites. The validity of this classification is supported by prior genomic audits of the TCGA database, which demonstrated over 98% concordance between self-identified race and genetically inferred ancestry, as determined via principal component analysis [21,22]. To ensure transparency and maintain statistical power, participants with “Unknown” or “Not Reported” status were retained in the primary cohort description.

### 2.3 Exposure Variables

Ethnicity was the primary exposure of interest. Clinical data associated with the publicly available TCIA GBM dataset were limited to White, African American, and Asian American cohorts due to insufficient sample sizes in other groups.

### 2.4 Covariates

Confounding variables evaluated in the multivariate analysis included age at diagnosis, sex, specific genomic alterations (e.g., chromosome +7/−10), and the Karnofsky Performance Status (KPS) [23]. Age was stratified into five categories: a baseline group of <50 years, followed by ten-year intervals extending up to 90 years (50–59, 60–69, 70–79, and 80–90). KPS was dichotomized into <70 and ≥70 (representing those able to perform self-care or normal activity). Unknown values were retained as a distinct category in the analysis.

### 2.5 Overall Survival Outcomes Assessment

The primary outcome was OS, defined as the time in months from diagnosis to all-cause mortality. Patients who were alive at the time of last contact were censored in the analysis. Survival outcomes were estimated using the Kaplan-Meier method, while log-rank tests and Cox proportional hazards regression models were used to evaluate associations with ethnicity, sex, and age. The interaction between chromosome +7/−10 co-occurrence and other variables was assessed using Cox proportional hazards models for OS and analyzed regarding GBM incidence rates. As an a priori review, the primary outcome was OS from age at initial pathological diagnosis, and the secondary outcome was 25 months of survival.

### 2.6 Statistical Analysis

The baseline clinical characteristics status and demographics of patients were compared using a Chi-square χ^2^ test for categorical variables and Kruskal-Wallis tests for continuous variables. OS was estimated using Kaplan-Meier methods associations between ethnicity and survival outcomes were evaluated with log-rank tests. Logistic regression with significance assessment by the likelihood ratio test was used to assess the association between ethnicity and genomic alteration overall and within types. Ethnic disparities in survival were evaluated using Cox proportional hazards regression, with White Americans designated as the reference cohort for hazard ratio (HR) calculations. The proportional hazards assumption was formally validated through the analysis of Schoenfeld residuals. Overall, within chromosome +7/−10, O^6^-methylguanine-DNA methyltransferase (MGMT) promoter methylation and the 1p/19q codeletion; significance was assessed using the Wald test. A *p* value < 0.05 was considered to be statistically significant. All analyses were conducted using R software version 4.3.1 (R Foundation for Statistical Computing, Vienna, Austria, https://www.R-project.org/) and SPSS software version 20.0 (IBM Corp, Armonk, NY, USA).

## 3 Results

### 3.1 Patient Population and Sensitivity Analysis

**Figure 2:**
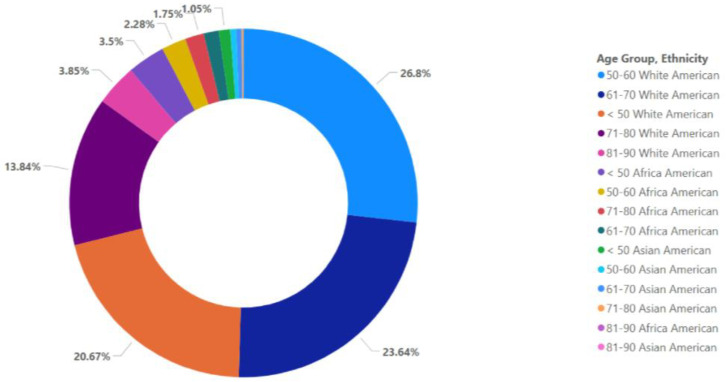
Age-specific distribution and demographic profile of the GBM cohort. The cohort was predominantly composed of White patients (89%), followed by African American (9%) and Asian American (2%) individuals.

Patient population of the 599 patients in the cohort, 571 were included in the association analysis for the primary endpoint of GBM, of whom 13 (2%) were identified as Asian, 51 (9%) as African, and 507 (89%) as White in epidemiology. The largest ethnic population with the most significant incidence rates is White-American, 26.8% [range, 50–60 years] (Fig. 2). The median age at diagnosis was distributed across ethnic groups (Asian patients: 52 years [range, 30–71 years]; African patients: 53 [range, 17–79 years]; White patients: 59 years [range, 17–89 years]) in Table 1.

**Figure 3:**
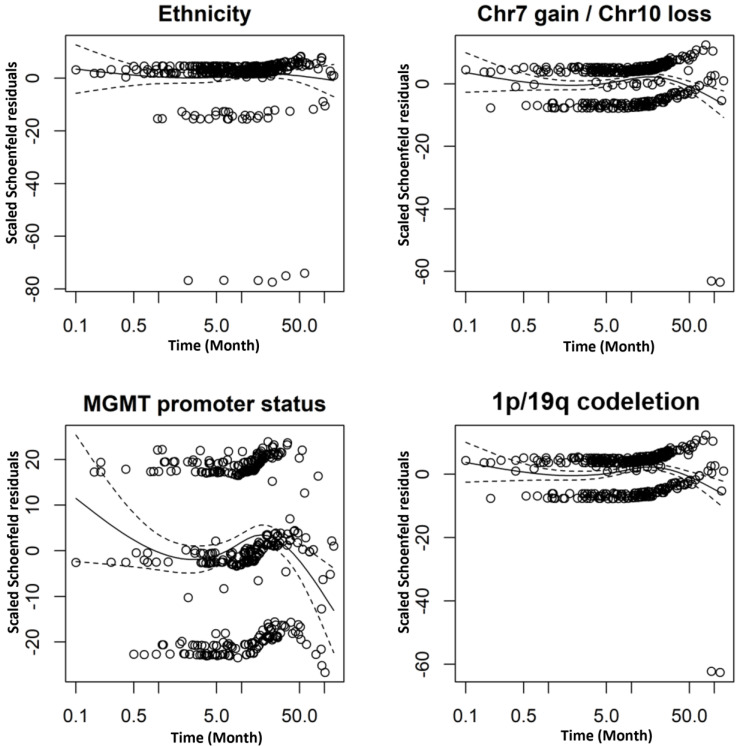
Validation of the proportional hazards assumption using Schoenfeld residual plots. The solid line depicts the estimated curve, representing time-dependent deviations from proportionality, while the dotted lines indicate the 95% confidence interval (CI).

The study leverages bioinformatics frameworks to analyze genomic and clinical data across diverse ethnic populations. Multivariate Cox proportional hazards analysis was employed to evaluate the independent associations between ethnicity, specific genetic alterations, and OS within the GBM cohort (Table 2). To quantify the impact of ethnicity on clinical outcomes, we employed a Cox proportional hazards model, calculating HR estimations relative to the White American reference group. The validity of the model fit and the proportional hazards assumption were confirmed through Schoenfeld residual testing (Fig. 3).

**Table 2:** Multivariate Cox proportional hazards model of GBM survival outcomes^a^.

Factors	No. of Patients, *n* (%)	HR^a^ (95% CI)	*p*-Value^b^
**Ethnicity**			
White American	507 (89%)	Ref (1.0)	
African American	51 (9%)	0.850 (0.578–1.250)	0.408
Asian American	13 (2%)	0.464 (0.206–1.047)	0.064
**Chromosome 7 gain/Chromosome 10 loss**			
No combined CNAs	357 (63%)	Ref (1.0)	
Chromosome +7/−10	187 (33%)	0.798 (0.647–0.984)	0.035*
Unknown	27 (4%)	-	-
**MGMT promoter methylation**			
Methylated	184 (32%)	Ref (1.0)	
Unmethylated	216 (38%)	1.180 (0.923–1.510)	0.187
Unknown	171 (30%)	1.087 (0.855–1.382)	0.495
**1p/19q codeletion**			
Codeletion	2 (0.3%)	Ref (1.0)	
Non-codeletion	548 (96%)	3.759 (0.916–15.436)	0.066
Missing	21 (3.7%)	-	-

Note: Bold denotes key variables identified for the sensitivity analysis, including ethnicity, chromosome 7 gain/chromosome 10 loss, MGMT promoter methylation, 1p/19q codeletion. ^a^This data adjusted for ethnicity, genetic alteration using multivariable Cox proportional hazards regression models. ^b^*p* < 0.05 (*) is considered as statistically significant. “-” coefficients do not converge in Cox regression statistical models. Chromosome +7/−10, chromosome 7 gain/Chromosome 10 loss; CNAs: copy number alterations; CI, confidence interval; GBM, glioblastoma; HR, hazard ratio; MGMT, O^6^-methylguanine-DNA methyltransferase; Ref, reference.

To address the robustness of the chromosome +7/−10 signature as a prognostic marker, we performed a sensitivity analysis to account for the potential statistical fragility of smaller minority cohorts (Table S1). Upon excluding the Asian American cohort (*n* = 13), the mortality risk associated with the chromosome +7/−10 signature remained remarkably stable (HR 0.802; 95% CI 0.649–0.991; *p* = 0.041) compared to the primary multivariate model. These results suggest that the primary genomic conclusions are not unduly biased by the limited sample sizes of underrepresented subgroups.

Furthermore, we conducted a formal interaction analysis to evaluate the relationship between ethnicity and genotype. However, due to sparse data within the minority cohorts (e.g., Asian population), several interaction terms were not estimable. This data sparsity further justifies the utilization of the main-effects model for our primary conclusions. Consequently, while the analysis of minority subgroups remains exploratory, the overarching association between the chromosome +7/−10 alteration and OS appears robust across the study population and quantitative comparison of survival differences across subgroups in the supplementary materials (Tables S2 and S3).

### 3.2 Tumor-Infiltrating Molecules within the TME Imaging Features

**Figure 4:**
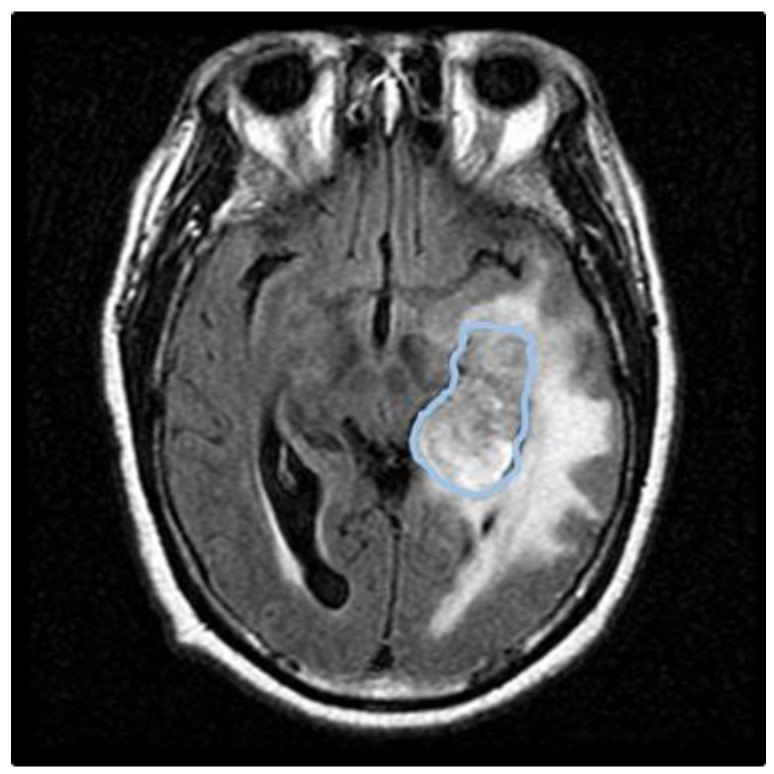
Axial fluid attenuated inversion recovery (FLAIR) MR Imaging. Left-hemisphere GBM illustrating tumor infiltration and vasogenic edema. Grey delineations identify peritumoral layers where immune-driven genetic markers and tumor-infiltrating lymphocytes enhance prognostic accuracy in survival modeling.

GBM is a highly aggressive malignancy defined by IDH-wildtype status, *EGFR* amplification, and *PTEN* loss. Its pathophysiology—characterized by rapid proliferation, necrosis, and angiogenesis—is sustained by a hypoxic niche where HIF-1α stabilization and immunosuppressive macrophages drive infiltrative growth along white matter tracts and vasculature. Crucially, MGMT promoter methylation and hallmark copy number variations shape the immune TME; our analysis reveals that TME composition corresponds to specific genetic and clinical endpoints (Table 2; Fig. 4). While glioma stem cells drive intratumoral heterogeneity and tumor initiation, the tumor actively suppresses infiltrating CD4^+^ and CD8^+^ T-cell activity. This interaction facilitates immune evasion, particularly within hypoxic zones, illustrating the complex interplay between molecular stratification and the peritumoral immune landscape.

### 3.3 Incidence and Mortality by Ethnicity

In a cohort study of 571 patients with GBM, baseline clinical characteristics (including age, sex at diagnosis, and KPS did not differ significantly across Asian, Black, and White American populations. However, genomic landscapes exhibited marked ethnic divergence; specifically, the prevalence of entire chromosome +7/−10 varied significantly between cohorts (Table 1). Regarding prognostic findings, Asian Americans demonstrated a 0.46-fold mortality risk compared to White Americans, a trend that reached marginal statistical significance. No significant association was observed between ethnicity and traditional predictive biomarkers, such as MGMT promoter methylation or 1p/19q codeletion (Table 2). Finally, regarding demographic trends, GBM incidence was higher in males aged 50–59 years (*n* = 105, 34%) than in females of the same age (*n* = 48, 24%) in this cohort. Additionally, sensitivity analyses were performed to confirm the stability of the 0.80-fold mortality risk associated with the chromosome +7/−10 genomic signature in both White and African American cohorts (Table S1).

### 3.4 Risk Stratification, Identify Genetic Predisposition Based on Biomolecule Markers by Ethnicity

**Figure 5:**
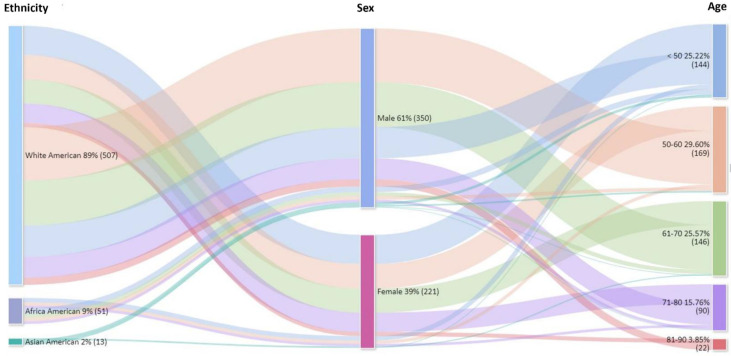
GBM incidence disparities by ethnicity, sex, and age. These patterns reveal a critical interplay between demographic factors and ancestry-specific genomic signatures, driving GBM variability across diverse populations.

Overall, White Americans constituted a significantly higher proportion of the cohort than other groups (*n* = 507, 89%). The expression of genomic alternative signatures was significantly higher in male patients (61% in males vs. 39% in females). Specifically, Patients aged 50–60 (*n* = 169, 29.60%) constituted the largest cohort in this population (Fig. 5). Among patients with GBM, three signatures in molecular markers chromosome +7/−10, MGMT promoter methylation, and 1p/19q codeletion were evaluated for differential expression across ethnic groups (Table 2), as these markers are predictive of therapeutic response. Of these three, only one chromosome +7/−10 signature was differentially expressed among ethnicities. This marker was associated with a significant survival benefit (HR 0.798; 95% CI 0.647–0.984; *p* = 0.035).

### 3.5 Associations between Ethnicity and Survival in Genomic Alteration

**Figure 6:**
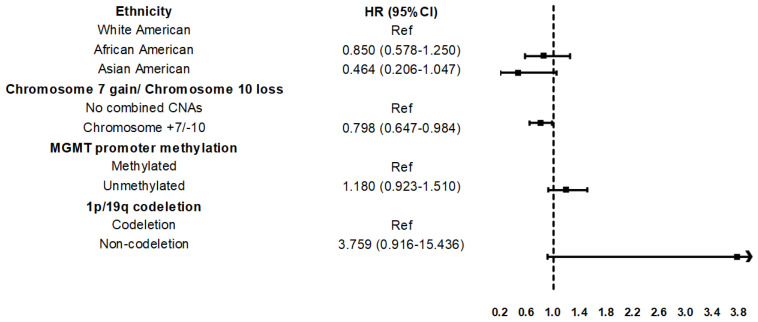
Forest plots illustrate the adjusted hazard ratios (HR) and 95% CI for each stratum. Stratified multivariate Cox regression analysis of GBM survival across clinical subgroups.

**Figure 7:**
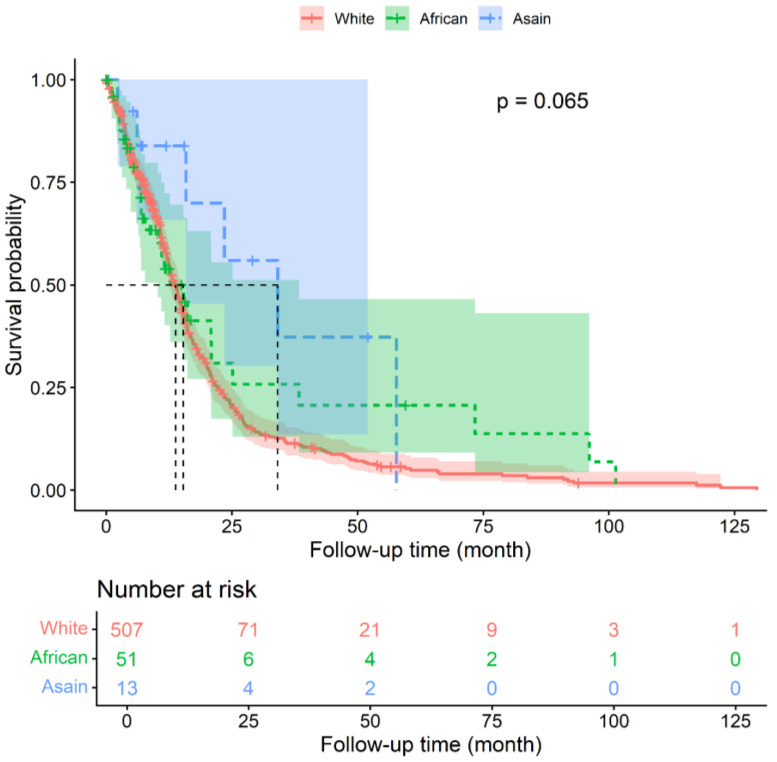
Kaplan–Meier survival analysis of GBM patients stratified by ethnicity. Cumulative survival functions illustrate the probability of overall survival across diverse ethnic cohorts.

Chromosome +7/−10 has significant ethnic differences with a higher mortality risk. The signature emerged as a significant independent prognostic factor, associated with a 20.2% reduction in mortality risk (HR 0.798; 95% CI 0.647–0.984; *p* = 0.035) relative to the no combined copy number alterations group. Significantly, this survival benefit was subject to ethnic stratification; while other clinical variables lost significance within specific strata, the prognostic value of the genomic profile remained robust. These associations, including potential survival disparities across diverse patient strata, are visualized via stratified forest plots (Fig. 6). These results highlight the stable prognostic value of ancestry-specific genomic signatures and ethnicity after adjusting for key clinical confounders. Among the enrolled patients, the cumulative incidence of the first death event within the first 25 months differed according to ethnicity: 71 White patients (14%), 6 African American patients (12%), and 4 Asian patients (31%), respectively in Fig. 7.

## 4 Discussion

In this retrospective cohort study, we compared the clinical outcomes of GBM in patients of different ethnicities, ages, and sex with clinical IDH wild-type GBM, genomic (molecular features based on chromosome +7/−10 as subtyping), chemoresistance (DNA repair enzyme MGMT) high-risk brain tumors in clinically meaningful investigation of this cohort studies. In this investigation of 571 patients, no significant differences in MGMT promoter methylation and 1p/19q codeletion were found among White, African, and Asian-American. Unexpectedly, we found that White Americans who carry chromosome +7/−10 had a higher mortality risk than their African and Asian-American counterparts. Exceptionally, White males have higher rates of GBM, however, we did not observe significant ethnic disparities in MGMT promoter methylation and 1p/19q codeletion outcomes within significant molecular signatures. Generally, MGMT promoter methylation, particularly when combined with 1p/19q codeletion, is a significant molecular feature associated with improved survival rate and response to alkylating chemotherapy such as Temozolomide [24,25,26,27]. Our findings suggest an association between ethnicity and chromosome +7/−10 in patients diagnosed with GBM, which is a crucial factor for significant indicators often associated with a specific molecular subtype and potentially poorer prognosis. This clinical significance is critical to understanding the genomic status of GBM, including substantial tumor progression in the 5th edition of the CNS classification on the chromosome +7/−10 profile, thereby forming a comprehensive investigation that integrates histology, immunohistochemistry, genetics, pathological data and advanced Magnetic Resonance (MR) imaging perspectives for physicians or radiologists to accurately aid in diagnosis, staging, and oncology-related predictive treatment decisions [7,14,28,29,30]. This investigation identified the existence of a difference in survival in GBM patients based on ethnicity such that African, Asian-American patients with GBM have better survival when compared to White.

American patients, after controlling for age, sex, and genetics. Despite the limited Asian American cohort available in public data repositories, we employed rigorous statistical thresholds and focused on high-confidence genomic alterations. Comparison the literature, including multi-institutional cohorts of up to 2390 patients [31] continues to show that African (3%) and Asian (5%) patients remain significantly underrepresented compared to White (92%) patients. These investigative findings expand on the topic of how ethnicity specifically impacts patient mortality by using a population more applicable to typical GBM patients. Consistent with previous studies, we found no significant difference in survival between White and African American GBM patients, in agreement with the results of most population-based African American studies [10,32,33,34]. Furthermore, a large population study from 2000 to 2014 appeared to confirm the reduced risk of mortality among Asian Americans in the United States [2,10]. Surprisingly, compared with some literature, our results reveal a possible reason why a large proportion of White Americans in different populations carry gain events of the entire chromosome 7 and loss events of chromosome 10, as well as the compensating gene enrichment analysis of specific chromosomes provides a plausible reason for this co-occurrence to occur preferentially in cancers originating from certain areas of the brain. Those tend to have worse OS, suggesting that the enhanced underlying tumor specifically affects GBM mortality in patients [35].

In the US cohort multigenerational population, there is a trend of younger age of diagnosis. Still, there were no significant ethnicity-driven differences in age of onset (59 years White, 53 years African, 52 years Asian). In contrast, the frequency of chromosome +7/−10 alteration rate (64% in Whites, 59% in Africans, and 31% in Asians) was significantly different among the groups. This research identified the existence of a difference in survival in GBM patients based on ethnicity such that African, Asian-American patients with GBM have better survival when compared to White-American patients, after controlling for age, sex, and ethnicity.

### 4.1 Molecular Biomarkers for Disease Diagnosis and Prognosis

In IDH-wildtype GBM, the genotypes defined by *EGFR* amplification (chromosome 7) and *PTEN* deletion (chromosome 10) exhibit distinct racial stratification. This stratification reveals a high frequency of the *EGFR*+/*PTEN*− signature in White patients, contrasting with a significantly lower prevalence in non-White populations. Specifically, White patients demonstrate a higher prevalence of *PTEN* alterations (48.67%) [31], validating the superior and advanced predictive utility of these standard biomarkers within Caucasian cohorts. This investigation expands on the critical role of ethnicity in GBM mortality by utilizing a cohort representative of typical patient demographics. Deep genomic profiling has profoundly advanced clinical classification through molecular subtyping, providing essential guidance for chemotherapy and precision medicine. GBM is a highly heterogeneous malignancy characterized by distinct microenvironments and molecular vulnerabilities, such as dysregulated NF-κB signaling, which contributes to aggressive invasion and therapeutic resistance. Molecular subtyping identifies these variations, leading to more accurate diagnoses and tailored targeted therapies [36,37,38]. Several studies have shown that phenotypic plasticity is a primary driver of intratumor heterogeneity and therapeutic resistance in GBM. This plasticity creates a dynamic tumor ecosystem in which diverse phenotypic states coexist, and interact with an evolving TME allowing the tumor to adapt to external pressure rapidly. Additionally, non-invasive omics approaches leverage translational radiomics imaging biomarkers (TRIB^®^) to integrate a triad of morphological, textural, and functional signatures, particularly, these imaging biomarkers reflect the phenotypic plasticity and key regulatory molecular shifts occurring within the TME [14,15,16,39,40]. More relevantly, by targeting specific molecular alterations, precision medicine can improve therapeutic efficacy and reduce toxicity, leading to better treatment outcomes amid challenges and future directions. Future prospective studies may investigate causes of the differences in survival between White, Asian and other ethnic groups. The association between *EGFR*, *TERT* promoter mutations and mortality could be studied independently of ethnicity, as this could be a key factor behind the differences we found. The results of this study can help improve the accuracy of the prognostic landscape for different ethnicities of patients with GBM. The implications of this study, these investigatory findings can help increase the accuracy of the diagnosis and poor prognostic prospects for White, African, and Asian-American patients with GBM on coverage continuity for the vulnerable population and genomic biomarkers across ethnicities. Notably, the prevalence of chromosome +7/−10 in White-American profile is higher than in Asian-American counterparts, which is a strong surrogate marker for the diagnosis of IDH-wildtype GBM. Particularly, when combined with other molecular markers such as *EGFR* gene amplification for tumor-specific aberrations, which hint at distinct carcinogenesis pathways. The co-occurrence of chromosome +7/−10 is a hallmark genetic signature of primary, IDH-wildtype GBM. Representing the most frequent aneuploidy pair in human GBM, this signature serves as a critical surrogate marker for WHO Grade 4 glioma. Biologically, chromosome 7 gain often involves the *EGFR* gene, while chromosome 10 loss typically results in the deletion of the *PTEN* tumor suppressor. Mechanistically, evidence suggests that the gain of chromosome 7 may function as a fitness compensation mechanism for the metabolic detriments induced by chromosome 10 loss, creating a synergistic environment for aggressive tumor proliferation and metabolic adaptation [35]. While our data confirm that the chromosome +7/−10 genotype predicts poor survival in White (Caucasian) cohorts, its lower prevalence in African and Asian American populations suggests the influence of ancestry-specific oncogenic drivers. Integrating such molecular stratification into diagnostic frameworks is essential for advancing biomarker-informed equity and ensuring that personalized GBM treatment strategies are effective across diverse ancestral populations. The TME is profoundly shaped by a core trio of genetic hallmarks: IDH-wildtype status, *EGFR* amplification, and *PTEN* loss. It reveals that these genomic features do not act in isolation but instead dictate the peritumoral immune landscape through specific mechanistic axes. We detail how the chromosome +7/−10 signature highly prevalent in White cohorts correlates with a profoundly immunosuppressive TME. Mechanistically, the synergy between chromosome +7/−10 drives HIF-1α stabilization and NF-κB signaling, pathways that actively recruit M2-polarized (pro-tumor) macrophages and exclude cytotoxic CD4^+^ and CD8^+^ T-cells. This genotype-to-immune coupling effectively fosters a cold tumor microenvironment, facilitating immune evasion [7,35,41,42,43]. This genomic-immune coupling provides a biological basis for the ancestry-specific differences observed in our study. The significantly lower prevalence of the chromosome +7/−10 signature in African and Asian American cohorts suggests that these populations may utilize divergent oncogenic drivers to modulate the TME. In these cohorts, other factors, such as MGMT promoter methylation status, appear to play a more dominant role in shaping the immune response and metabolic adaptation. By identifying these distinct TME profiles, our findings highlight how ethnic variations in genomic alterations lead to unique immune evasion strategies, reinforcing the need for ancestry-informed molecular stratification in personalized GBM diagnostics.

### 4.2 Strengths and Limitations

The scientific merit of this study lies in identifying chromosome +7/−10 alterations as a key independent prognostic factor for poor survival in White (Caucasian) males with GBM. This clinical outcome is driven by a hypoxic niche, where HIF-1α stabilization and immunosuppressive macrophages propel infiltrative growth along white matter tracts and vasculature. While the epidemiological incidence of these markers is lower in African American and Asian American populations, our findings highlight the importance of biomarker-based diagnostics across diverse ancestries. However, this study has several limitations. First, the survival comparisons for minority subgroups are statistically fragile due to the small sample sizes of African American (*n* = 51) and Asian American (*n* = 13) participants. Consequently, these specific findings are exploratory and were not powered to yield definitive conclusions. Second, exclusion criteria removed 28 patients with incomplete clinical or genomic data, which may introduce selection bias. Third, the lack of comprehensive genetic assessment in the Asian American cohort hindered a broader comparison of diverse genomic alterations across ethnicities. Notably, in contemporary multi-institutional cohorts of 2390 patients, White patients (92%) overwhelmingly dominate, while African (3%) and Asian (5%) populations remain critically underrepresented [31]. Given this scarcity, our analysis provides a vital albeit exploratory foundation. Future prospective studies must expand these population-based approaches to mitigate statistical fragility and better characterize GBM genomic landscapes in global populations. Furthermore, while our analysis focused on the chromosome +7/−10 signature, we acknowledge that other molecular drivers, such as *EGFR* amplification and *TERT* promoter mutations, also play vital roles in GBM progression. These markers were excluded from our multivariate analysis due to incomplete data across the ethnic cohorts in the public repository. Subsequent studies integrating broader genomic profiles will be essential to map the complex relationship between ancestry-specific alterations and survival. Most pertinently, the limitation of this study is the lack of detailed data on socioeconomic status, insurance coverage, and treatment-specific factors such as the extent of resection and adjuvant therapy regimens, including immunotherapy or targeted therapy. Despite these limitations, our findings provide significant insights into the prognostic impact of ethnicity in patients with GBM, highlighting potential disparities that warrant further investigation in prospective trials. As these factors are critical drivers of GBM survival that often vary by ethnicity, their absence in current datasets may confound the observed survival disparities. Consequently, the identified chromosome +7/−10 signature should be viewed as only one component within a broader, multifactorial landscape of disparity. To mitigate the risk of genetic determinism, we emphasize that our findings must be interpreted through the lens of structural healthcare inequities rather than as a singular argument for biological drivers. The observed survival differences likely reflect a complex interplay between ancestry-specific molecular landscapes and unmeasured social determinants, highlighting the need for future research that integrates clinical genomics with comprehensive social health data.

## 5 Conclusion

This retrospective cohort study establishes that risk factors and surrogate biomarkers associated with GBM may identify crucial ethnic disparities. Our bioinformatics analysis confirms that the chromosome +7/−10 genotype is a hallmark of poor survival in Caucasian males with high-risk GBM. However, its lower prevalence in African and Asian American cohorts suggests distinct, ancestry-specific oncogenic drivers. While these molecular signatures are vital for biomarker-informed diagnostics, they must be interpreted within the broader context of healthcare equity such as socioeconomic status and treatment access. Ultimately, personalized GBM strategies must integrate molecular stratification with the mitigation of systemic barriers to effectively address survival disparities across diverse populations.

## Data Availability

De-identified clinical and imaging data were retrieved from the TCGA-GBM collection via The Cancer Imaging Archive (TCIA), a publicly accessible repository for the research community.

## Supplementary Materials

The supplementary material is available online at https://www.techscience.com/doi/10.32604/or.2026.077076/s1.
