# Supplementary material for: Ethnic Disparities in Glioblastoma Markers: Impact of Chromosome 7 Gain and 10 Loss Alterations on Clinical Survival Outcomes
Source: Oncol Res. 2026 May 21;34(6):21. doi: 10.32604/or.2026.077076 (PMC13223225; doi:10.32604/or.2026.077076)
Supplement: Supplementary file 1 [file OncolRes-34-77076-s001.zip › TSP_OR_77076-s001.docx]

**Supplementary Tables**

**Table S1**: Multivariate Cox Proportional Hazards Model for Sensitivity Analysis of GBM Survival Predictors

| **Factors** | **No. of patients** | **HR^a^ (95% CI)** | ***p* -value^b^** |
| --- | --- | --- | --- |
| **Ethnicity** |  |  |  |
| White American | 507 (91%) | Ref (1.0) |  |
| African American | 51 (9%) | 0.850 (0.578-1.250) | 0.408 |
| **Chromosome 7 gain/ Chromosome 10 loss** |  |  |  |
| No combined CNAs | 353 (63%) | Ref (1.0) |  |
| Chromosome +7/-10 | 178 (32%) | 0.802 (0.649-0.991) | 0.041* |
| Unknown | 27 (5%) | ─ | ─ |
| **MGMT promoter methylation** |  |  |  |
| Methylated | 176 (32%) | Ref (1.0) |  |
| Unmethylated | 214 (38%) | 1.197 (0.934-1.534) | 0.155 |
| Unknown | 168 (30%) | 1.109 (0.870-1.414) | 0.404 |
| **1p/19q codeletion** |  |  |  |
| Codeletion | 2 (0.3%) | Ref (1.0) |  |
| Non-codeletion | 535 (96%) | 3.722 (0.906-15.287) | 0.068 |
| Missing | 21 (3.7%) | ─ | ─ |

Note: ^a^ This data adjusted for ethnicity, genetic alteration and age at diagnosis using multivariable Cox proportional hazards regression models. ^b^ *p* < 0.05(*) is considered as statistically significant. “─” coefficients do not converge in Cox regression statistical models. Chromosome +7/-10, chromosome 7 gain/ Chromosome 10 loss; CI, confidence interval; CNAs, copy number alterations; GBM, glioblastoma; HR, hazard ratio; MGMT, O^6^-methylguanine-DNA methyltransferase; Ref, reference.

**Quantitative Comparison of Survival Differences across Subgroups**

| **Factors** | **No. of patients** | **HR^a^ (95% CI)** | ***p* -value** |  |
| --- | --- | --- | --- | --- |
| **Ethnicity** |  |  |  |  |
| White American | 323 (91%) | Ref (1.0) |  |  |
| African American | 30 (8%) | 0.678 (0.416-1.106) | 0.119 |  |
| Asian American | 4 (1%) | 0.462 (0.114-1.879) | 0.280 |  |
| **MGMT promoter methylation** |  |  |  |  |
| Methylated | 102 (29%) | Ref (1.0) |  |  |
| Unmethylated | 144 (40%) | 1.230 (0.898-1.684) | 0.198 |  |
| Unknown | 111 (31%) | 1.078 (0.789-1.472) | 0.639 |  |
| **1p/19q codeletion** |  |  |  |  |
| Codeletion | 0 (0%) | Ref (1.0) |  |  |
| Non-codeletion | 357(100%) | None | None |  |
| Missing | 0 (0%) | ─ | ─ |  |

**Table S2:** Multivariate Cox Regression Analysis of GBM Survival by Ethnicity and Genomic CNA Profile

Note: ^a^ This data adjusted for ethnicity, genetic alteration and age at diagnosis using multivariable Cox proportional hazards regression models. “─” coefficients do not converge in Cox regression statistical models. Chromosome +7/-10, chromosome 7 gain/ Chromosome 10 loss; CI, confidence interval; CNAs, copy number alterations; GBM, glioblastoma; HR, hazard ratio; MGMT, O^6^-methylguanine-DNA methyltransferase; Ref, reference.

**Quantitative Comparison of Survival Differences across Subgroups**

**Table S3:** Multivariate Cox Regression Analysis of GBM Survival by Ethnicity and Chromosome +7/-10 Profile

| **Factors** | **No. of patients** | **HR^a^ (95% CI)** | ***p* -value** |  |
| --- | --- | --- | --- | --- |
| **Ethnicity** |  |  |  |  |
| White American | 164 (88%) | Ref (1.0) |  |  |
| African American | 14 (7%) | 1.379 (0.708-2.683) | 0.345 |  |
| Asian American | 9 (5%) | 0.479 (0.176-1.306) | 0.150 |  |
| **MGMT promoter methylation** |  |  |  |  |
| Methylated | 72 (38%) | Ref (1.0) |  |  |
| Unmethylated | 63 (34%) | 1.116 (0.735-1.694) | 0.605 |  |
| Unknown | 52 (28%) | 1.044 (0.700-1.557) | 0.833 |  |
| **1p/19q codeletion** |  |  |  |  |
| Codeletion | 2 (1%) | Ref (1.0) |  |  |
| Non-codeletion | 185 (99%) | 3.626 (0.861-15.268) | 0.079 |  |
| Missing | 0 (0%) | ─ | ─ |  |

Note: ^a^ This data adjusted for ethnicity, genetic alteration and age at diagnosis using multivariable Cox proportional hazards regression models. “─” coefficients do not converge in Cox regression statistical models. Chromosome +7/-10, chromosome 7 gain/ Chromosome 10 loss; CI, confidence interval; CNAs, copy number alterations; GBM, glioblastoma; HR, hazard ratio; MGMT, O^6^-methylguanine-DNA methyltransferase; Ref, reference.
